# RAPID-CARE: Rapid Antibiotic Optimization in the ICU After Implementation of a Pneumonia Multiplex PCR Test—A Real-World Evaluation

**DOI:** 10.3390/antibiotics14111084

**Published:** 2025-10-27

**Authors:** Montserrat Rodríguez-Gómez, Fernando Martínez-Sagasti, María Calle-Romero, Andrea Prieto-Cabrera, Patricia De La Montaña-Díaz, Irene Díaz-De la Torre, Alberto Delgado-Iribarren García-Campero, Sara Domingo-Marín, Miguel Sánchez-García, Ignacio Martín-Loeches

**Affiliations:** 1 Department of Intensive Care Medicine, University Hospital Clínico San Carlos, 28040 Madrid, Spainapcabrera@salud.madrid.org (A.P.-C.);; 2 Department of Clinical Microbiology, University Hospital Clínico San Carlos, 28040 Madrid, Spain; irene.diazdela@salud.madrid.org (I.D.-D.l.T.);; 3 St James’s University Hospital, Trinity College, D08 NHY1 Dublin, Ireland

**Keywords:** multiplex PCR, BioFire^®^ FilmArray^®^ Pneumonia Panel Plus, hospital-acquired pneumonia, ventilator-associated pneumonia, ventilator-associated tracheobronchitis (VAT), antimicrobial stewardship, intensive care unit, rapid molecular diagnostic test

## Abstract

**Background/Objectives**: Lower respiratory tract infections (LRTIs) are frequent in the intensive care unit (ICU) and drive empiric broad-spectrum antibiotic use. Rapid multiplex PCR assays may improve pathogen detection and stewardship compared with conventional culture. We evaluated the real-world impact of the BioFire^®^ FilmArray^®^ Pneumonia Panel Plus (FA-PNEU^®^) on antimicrobial management in suspected nosocomial LRTI. **Methods**: This was a single-centre, prospective observational cohort study conducted in a tertiary ICU (Madrid, Spain) between April 2021 and March 2025. Adult patients with suspected hospital-acquired pneumonia (HAP), ventilator-associated pneumonia (VAP), or ventilator-associated tracheobronchitis (VAT) were included if paired respiratory samples underwent FA-PNEU^®^ and conventional culture (CC). Diagnostic accuracy and prescribing changes were analysed. **Results**: A total of 344 samples from 236 patients were included. FA-PNEU^®^ demonstrated high sensitivity (93.4%) and negative predictive value (97.9%) but moderate specificity (65.0%) and low positive predictive value (36.5%). False positives occurred in 85.8% of patients with prior antibiotic therapy targeting the detected organism. Antibiotic management was considered directly influenced by FA-PNEU^®^ when any prescribing decision (initiation, escalation, de-escalation, or discontinuation) explicitly followed the panel’s results rather than other clinical or microbiological information. Using this definition, FA-PNEU^®^ directly influenced antibiotic therapy in 57.6% of cases, while in 17.7%, prescribing was instead guided by a suspected alternative infection. In patients without prior antibiotics, treatment initiation or withholding was fully concordant with FA-PNEU^®^ results, while in those already receiving therapy, 60.8% underwent modification, two-thirds in agreement with the panel. **Conclusions**: In critically ill patients with suspected nosocomial LRTI, FA-PNEU^®^ provided rapid, high-sensitivity diagnostics that substantially influenced antimicrobial prescribing. Its greatest value lies in ruling out bacterial infection and guiding stewardship, though results must be interpreted within the full clinical and microbiological context.

## 1. Introduction

Lower respiratory tract infection (LRTI) is both a frequent reason for intensive care unit (ICU) admission [1] and a common complication among critically ill patients [2]. In the 2017 International Study of Prevalence and Outcomes of Infection in the ICU, LRTI was identified as the most common source of infection globally among ICU patients [3]. In the ICU setting, LRTIs can manifest as hospital-acquired pneumonia (HAP), occurring more than 48 h after hospital admission, or ventilator-associated pneumonia (VAP), occurring more than 48 h after initiation of invasive mechanical ventilation (IMV) [4]. Another related condition, ventilator-associated tracheobronchitis (VAT), presents with fever and increased purulent secretions in patients under IMV without radiographic evidence of pneumonia. The limited sensitivity and specificity of chest radiography often makes it difficult to distinguish between VAT and VAP, and diagnostic uncertainty is common [5].

Timely and appropriate antimicrobial therapy is critical in LRTI, as delays in effective treatment have been repeatedly associated with increased mortality [6,7]. For HAP and VAP, international guidelines recommend immediate empirical broad-spectrum antimicrobial therapy [8]. In VAT, early and appropriate antibiotic initiation has been associated with reduced progression to VAP [9]. As a result, many critically ill patients with suspected LRTI receive empiric regimens active against *Pseudomonas* spp. and frequently against methicillin-resistant *Staphylococcus aureus* (MRSA), even though cultures may later reveal the absence of multidrug-resistant organisms. This empiricism is complicated further when the clinical presentation could be explained by alternative sources of infection or non-infectious causes, making targeted therapy more difficult.

Rapid molecular diagnostics, such as the BioFire^®^ FilmArray^®^ Pneumonia Panel (FA-PNEU^®^), can detect a wide range of respiratory pathogens and resistance genes within approximately one hour, providing an opportunity to optimise antimicrobial therapy by enabling earlier de-escalation or escalation compared to conventional culture (CC) methods [10]. In 2021, the FA-PNEU^®^ was introduced into our institution’s microbiology portfolio. At that time, ICU protocol for suspected LRTI followed Infectious Diseases Society of America (IDSA) guidelines, which recommended respiratory and blood cultures plus immediate empiric broad-spectrum therapy pending results. Given the potential of FA-PNEU^®^ to improve the appropriateness and timeliness of antibiotic prescribing in real-world ICU practice, we hypothesised that access to FA-PNEU^®^ results would lead to measurable changes in antibiotic management for patients with suspected LRTI, compared to decisions made without such rapid diagnostic input. Our main research question was as follows: In critically ill patients with suspected LRTI, how does access to FA-PNEU^®^ results influence antimicrobial prescribing decisions compared to standard practice based on conventional culture and clinical judgment?

Despite multiple evaluations of the FA-PNEU^®^ in controlled or laboratory settings, most prior studies have focused on diagnostic performance rather than its practical influence on bedside decision-making. Data on how FA-PNEU^®^ results translate into real-world antibiotic prescribing, particularly within high-intensity ICU environments, where empiric therapy is almost universal, remain limited. This study addresses that gap by prospectively quantifying the direct impact of FA-PNEU^®^ on antimicrobial management and contextualizing its added value alongside conventional microbiology and clinical reasoning in a real-world critical care setting.

## 2. Results

**Figure 1:**
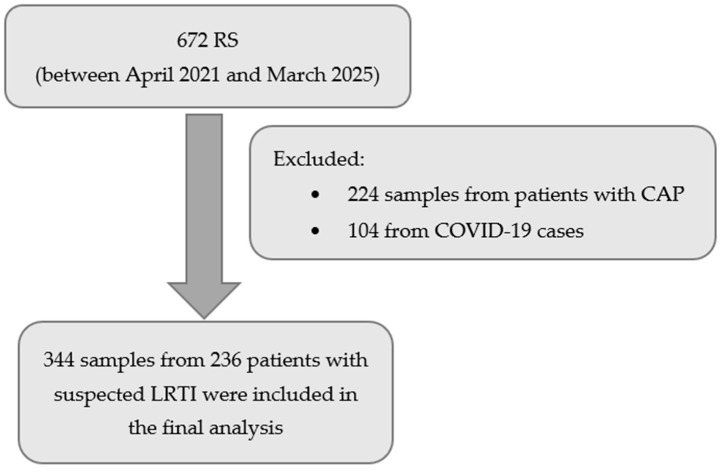
Study flowchart. RS: respiratory secretion; CAP: community-acquired pneumonia; LRTI: Lower respiratory tract infection.

Between April 2021 and March 2025, 672 respiratory secretion (RS) samples were processed in the ICU. After excluding 224 samples from patients with community-acquired pneumonia and 104 from COVID-19 cases, 344 samples from 236 patients with suspected LRTI were included in the final analysis (Figure 1).

Patient characteristics are shown in Table 1. The mean age was 62.7 years (SD ±0.12), and 66.9% were male. The mean APACHE II score at admission was 20.9 (SD ±0.33). The most frequent reasons for ICU admission were respiratory failure (33.9%), postoperative cardiac or vascular surgery (17.4%), and septic shock (11.9%). ICU mortality was 28.8%, slightly below the 40% predicted by APACHE II.

At the sample level (Table 2), hospital-acquired pneumonia (HAP) accounted for 49.4% of suspected cases, ventilator-associated tracheobronchitis (VAT) for 31.1%, and ventilator-associated pneumonia (VAP) for 19.5%. Tracheal aspirates were the most common specimen type (68.3%), and 86.0% of samples were from patients with multidrug-resistant (MDR) risk factors.

When comparing the BioFire^®^ FilmArray^®^ Pneumonia (FA-PNEU^®^) Panel Plus with conventional culture (CC) (Table 3), FA-PNEU^®^ demonstrated a sensitivity of 93.4% (95% CI, 84.1–98.2; 57/61), specificity of 65.0% (95% CI, 59.4–70.3; 184/283), positive predictive value (PPV) of 36.5% (95% CI, 29.1–44.5; 57/156), and negative predictive value (NPV) of 97.9% (95% CI, 94.7–99.2; 184/188). Notably, 85.8% of false positives occurred in patients with prior antibiotic therapy targeting the detected organism.

Concordance analysis (Table 4) showed complete agreement between FA-PNEU^®^ and CC in 57.0% of samples. Incomplete concordance occurred in 43.0% of cases, most commonly due to additional organisms detected by FA-PNEU^®^ (*n* = 104) compared to CC (n = 23).

The frequency of organism detection differed between methods (Table 5). For example, *Haemophilus influenzae* was detected in 10.1% of FA-PNEU^®^ samples but only 3.9% of CC samples, while *Serratia marcescens* was more frequent in CC (20.6%) than FA-PNEU^®^ (9.5%). Several viral pathogens were identified exclusively by FA-PNEU^®^.

Resistance mechanisms detected by FA-PNEU^®^ and CC are presented in Table 6. FA-PNEU^®^ identified more *bla*_VIM_-positive isolates (45.8%) compared with CC (41.2%). Other carbapenemase genes (*bla*_NDM_, *bla*_OXA-48_, *bla*_IMP_) were absent in both methods. Detection of *bla*_mecA/mecC_ and *bla*_MREJ_ genes was comparable (25.0% vs. 29.4%).

Organisms outside the FA-PNEU^®^ panel but recovered by CC are listed in Table 7, including *Aspergillus *spp. (27.3%), *Stenotrophomonas maltophilia* (20.5%), and *Enterococcus *spp. (15.9%), many of which are not traditional pneumonia pathogens but may be relevant in immunocompromised ICU populations.

Temporal trends (Table 8) showed progressive adoption of FA-PNEU^®^ from 2021 to 2024, peaking at 9.8 samples/month.

Reasons for antibiotic modification (Table 9) were most frequently based directly on FA-PNEU^®^ results (57.6%), followed by suspicion of an alternative infectious focus (17.7%).

Real-world antibiotic management (Table 10) showed that among patients without prior antibiotics (n = 89), therapy initiation (n = 38) or withholding (n = 51) was fully concordant with FA-PNEU^®^ results. In those already receiving antibiotics (n = 255), 100 cases (39.2%) had no therapy change, while 155 (60.8%) underwent modification—69.6% of which were concordant with FA-PNEU^®^ data.

## 3. Discussion

In this single-centre, real-world evaluation of the BioFire^®^ FilmArray^®^ Pneumonia (FA-PNEU^®^) panel for suspected nosocomial lower respiratory tract infections (LRTI) in critically ill patients, we observed that access to rapid multiplex PCR results had a substantial impact on antimicrobial prescribing, with changes or decisions directly attributable to the assay in 57.6% of cases. This aligns with prior studies reporting rapid molecular diagnostics as powerful antimicrobial stewardship tools, capable of supporting earlier escalation, targeted therapy, or de-escalation in high-risk ICU populations [11,12,13].

### 3.1. Diagnostic Performance and Concordance with Culture

FA-PNEU^®^ demonstrated high sensitivity (93.4%) and an excellent negative predictive value (NPV, 97.9%), making it particularly useful for ruling out bacterial pathogens in suspected LRTI. However, specificity was moderate (65.0%) and the positive predictive value (PPV) was low (36.5%). The low PPV, largely driven by prior antibiotic therapy in 85.8% of false positives, is consistent with previous observations that molecular methods may detect non-viable bacterial DNA in patients already receiving antibiotics.

From a clinical perspective, interpreting these apparent false positives requires integrating microbiological findings with the patient’s trajectory and inflammatory response. In pretreated patients, FA-PNEU^®^ detections may represent residual DNA from effectively treated pathogens or colonization rather than ongoing infection. Distinguishing between these scenarios is essential to avoid unnecessary escalation. A practical framework involves combining FA-PNEU^®^ results with quantitative bacterial load, radiographic evolution, and biomarkers such as C-reactive protein or procalcitonin. When bacterial load is low and inflammatory markers are falling, results should be interpreted as colonization or microbiological residue rather than active infection. Conversely, high bacterial load or persistently elevated biomarkers despite prior therapy should prompt reassessment for treatment failure or superinfection. Embedding such an interpretative algorithm into stewardship discussions ensures FA-PNEU^®^ is used to refine, rather than expand, antimicrobial coverage in complex ICU cases.

Lee et al. reported that the BioFire^®^ FilmArray^®^ Pneumonia Panel had strong concordance for common Gram-negative pathogens but frequently detected additional organisms in patients with prior antibiotic exposure, many of which were of uncertain clinical relevance [14]. Similarly, Yoo et al. observed excellent NPV for Gram-negative pathogens but noted that polymicrobial detections were more frequent compared to culture, raising questions about interpretation in pre-treated patients [15].

The clinical utility of multiplex PCR testing is therefore highly dependent on pretest probability. In patients with low suspicion of LRTI, the high NPV of FA-PNEU^®^ allows clinicians to confidently rule out bacterial pneumonia and redirect diagnostic efforts toward alternative sources. In contrast, in patients with a high pretest probability, a positive FA-PNEU^®^ result may support rapid, targeted antimicrobial therapy, while a negative result should prompt clinicians to consider pathogens not represented in the panel.

Concordance analysis revealed complete agreement with conventional culture in 57.0% of cases. FA-PNEU^®^ detected additional organisms more often than culture (104 vs. 23), consistent with findings from Buchan et al., who reported higher pathogen detection with FA-PNEU^®^ (90% vs. 66% for culture) in hospitalized pneumonia patients, though often at lower bacterial loads [16]. Murphy et al. also confirmed the analytical performance of FA-PNEU^®^ across multiple centers and highlighted the importance of bacterial load thresholds in interpreting results [10]. The added detection of viral pathogens by FA-PNEU^®^ further increases its clinical relevance, enabling adjunctive antiviral therapy or avoiding unnecessary antibacterial use.

The INHALE WP3 programme has provided further insight into the clinical application of FA-PNEU^®^. The published trial protocol [17] outlined a pragmatic, multicenter RCT comparing PCR-guided versus standard care in ICU patients with HAP/VAP. A subsequent cost-effectiveness analysis reported reduced ICU costs (£33,149 vs. £40,951; –£7802 difference) and stewardship benefits with FA-PNEU^®^, but no improvement in clinical cure rates [18]. This reinforces the recurring theme that the strongest contribution of FA-PNEU^®^ lies in optimizing early prescribing and antimicrobial stewardship, while its direct effect on hard clinical outcomes remains uncertain.

### 3.2. Organism and Resistance Detection

The organism detection profile differed notably between FA-PNEU^®^ and conventional culture. Some Gram-negative bacteria, such as *Haemophilus influenzae*, were more frequently identified by FA-PNEU^®^, whereas others, including *Serratia marcescens*, were more commonly detected in culture. This discrepancy likely reflects differences in assay design (i.e., specific genetic targets included in the multiplex panel), analytical sensitivity thresholds, and pre-analytical factors such as sample quality and handling. Importantly, FA-PNEU^®^ is limited by its closed target list: clinically relevant pathogens such as *Aspergillus *spp. and *Stenotrophomonas maltophilia* are not represented in the panel. This limitation may reduce its standalone utility in immunocompromised populations or in patients at risk of infections caused by atypical or emerging pathogens [19]. Our previous studies have repeatedly emphasized this gap, particularly in the setting of ventilator-associated pneumonia (VAP), where broad microbiological coverage and careful integration with clinical context are essential for accurate diagnosis and antimicrobial stewardship [20,21].

With regard to resistance genes, FA-PNEU^®^ detected a greater number of carbapenemase-producing organisms than standard culture, especially *bla*_VIM_ positive isolates, underscoring its potential value for infection control and early containment strategies. This rapid recognition of multidrug-resistant Gram-negative organisms is particularly relevant in the ICU, where timely initiation of appropriate therapy strongly influences outcomes. However, it is critical to recognize that carbapenem resistance in *Pseudomonas aeruginosa* often results from mechanisms outside the scope of the panel (e.g., efflux pump upregulation, porin loss), meaning that phenotypic susceptibility testing remains indispensable. In our own work, we have shown that reliance on molecular platforms alone may over- or under-estimate resistance profiles, and that a hybrid approach combining rapid diagnostics with confirmatory phenotypic testing best supports clinical decision-making and stewardship goals [22,23].

Taken together, while FA-PNEU^®^ offers an important step forward in rapid pathogen identification and resistance gene detection, its integration into clinical care must be guided by an understanding of its limitations, complemented by culture, and interpreted within the broader clinical context—an approach consistently advocated in our research on ICU pneumonia diagnostics and management.

### 3.3. Impact on Antimicrobial Management

FA-PNEU^®^ results prompted a wide range of antibiotic decisions. In patients without prior antibiotic exposure, initiation or withholding of therapy was fully concordant with panel findings, suggesting strong clinician confidence in negative results. In contrast, among patients already on antibiotics, adherence to panel guidance was lower (69.6% in those with therapy changes, 30.4% in those without). This discrepancy likely reflects both the difficulty of de-escalating or discontinuing antimicrobials in hemodynamically unstable patients and the integration of other diagnostic information, such as imaging or inflammatory biomarkers, into clinical reasoning.

Notably, 17.7% of antibiotic changes were triggered by suspicion of an alternative infectious source following a negative FA-PNEU^®^ result, illustrating the assay’s indirect diagnostic value. This “rule-out” function has been less frequently emphasised in prior evaluations, but is particularly relevant in critically ill patients, where diagnostic uncertainty is common. Similar findings have been reported in other cohorts.

Collectively, these studies suggest that while FA-PNEU^®^ can directly shape initial prescribing, its greatest added value may lie in its ability to provide rapid reassurance in cases where pneumonia is not supported, thereby redirecting attention toward alternative infectious or non-infectious diagnoses. This aligns with stewardship principles recently reinforced in ICU pneumonia management guidelines [24].

### 3.4. Cost-Effectiveness Evidence from Randomised Data

Our findings regarding antibiotic stewardship and rapid decision-making are supported by evidence from the INHALE WP3 pragmatic multi-centre RCT, which compared BioFire^®^ FilmArray^®^ Pneumonia Panel-guided therapy with standard care in ICU patients with HAP or VAP [17]. In this trial, PCR-guided therapy achieved superior antibiotic stewardship outcomes at 24 h and was associated with lower mean ICU costs (£33,149 vs. £40,951; −£7802 difference) [18], despite the cost of the assay. Importantly, while the intervention was cost-effective for stewardship outcomes, it did not demonstrate superiority for clinical cure at 14 days, with fewer cases classified as clinically cured in the PCR arm [25,26]. This reflects a potential trade-off also observed in our real-world setting: FA-PNEU^®^ supports earlier, more precise stewardship decisions, but its effect on hard clinical outcomes may be blunted unless results are embedded within clear clinical pathways.

The INHALE data align with findings from other evaluations of syndromic PCR in lower respiratory tract infections. For example, Timbrook et al. [11] reported that rapid multiplex PCR testing reduced time to appropriate therapy and enabled de-escalation, though stewardship impact varied by setting and clinician adoption. Similarly, Buchan et al. found that BioFire^®^ FilmArray^®^ Pneumonia Panel improved pathogen detection compared to culture and influenced antibiotic prescribing, particularly by reducing unnecessary broad-spectrum coverage [16].

These converging data reinforce the concept that the principal, consistent benefit of syndromic PCR platforms in ICU pneumonia is optimization of early antimicrobial prescribing. By rapidly ruling in or out common pathogens and key resistance genes, these assays can reduce unnecessary exposure to carbapenems and other broad-spectrum agents, thereby lowering costs and potentially limiting resistance selection pressure. However, translating these microbiological and stewardship gains into improved clinical outcomes such as mortality, length of stay, or cure rates appears to require additional steps—specifically, the integration of diagnostics into multidisciplinary stewardship programmes, structured decision algorithms, and ongoing clinician training. This has been highlighted in pneumonia guideline commentaries; which emphasize that molecular diagnostics should be embedded within stewardship frameworks rather than used in isolation [27].

### 3.5. Strengths and Limitations

Strengths of this study include its real-world setting, sizeable sample over a four-year period, and integration of microbiological and clinical decision-making data. This provides an authentic picture of how FA-PNEU^®^ is used outside a research protocol and how it influences antimicrobial stewardship.

Limitations include its single-centre design, which may limit generalisability, and the observational nature of the study, which precludes definitive attribution of patient outcomes to FA-PNEU^®^ use. The absence of standardised protocols for interpreting FA-PNEU^®^ results also means that management decisions were subject to individual clinician judgement. Furthermore, the study did not assess cost-effectiveness or long-term outcomes such as antibiotic days, resistance emergence, or mortality.

### 3.6. Clinical Implications and Future Directions

Our findings, together with the INHALE WP3 trial results, suggest that FA-PNEU^®^ can be a valuable adjunct to conventional culture in managing nosocomial LRTI in the ICU, particularly for rapid pathogen exclusion, early resistance detection, and stewardship optimisation. Its clinical and economic impact is greatest when incorporated into structured diagnostic and stewardship frameworks. Future multicentre trials should evaluate patient-centred outcomes, cost–benefit ratios, and implementation strategies that ensure results are acted upon consistently and rapidly.

## 4. Materials and Methods

### 4.1. The Study Design and Setting

This was a single-centre, prospective observational cohort study conducted in the general ICU of Hospital Clínico San Carlos, Madrid, Spain, between April 2021 and March 2025. The ICU admits mixed medical and surgical critically ill patients. The study was approved by the Institutional Review Board (protocol number 23/454-O_P).

### 4.2. Participants

We included adult patients (≥18 years) admitted to the ICU in whom LRTI was clinically suspected during ICU stay and for whom a respiratory secretion (RS) sample was sent for both FA-PNEU^®^ testing and standard bacterial culture with susceptibility testing. LRTI suspicion was based on at least one clinical sign (fever or hypothermia, leukocytosis or leukopenia, purulent respiratory secretions, increasing oxygen requirement) combined with new or worsening pulmonary infiltrates on chest radiograph, or clinical suspicion of VAT without radiographic infiltrates. Patients were excluded if they had incomplete microbiological data, if FA-PNEU^®^ was performed without paired culture, or if death occurred before results could be acted upon.

### 4.3. Procedures

At the time of suspected LRTI, the standard diagnostic protocol included the following:

1. Collection of RS for both FA-PNEU^®^ and CC.
2. Blood cultures.
3. Additional samples (e.g., urine, wound swabs) if alternative infection sources were suspected.

The FA-PNEU^®^ results were made available to the treating ICU team as soon as testing was complete, including organism identification and resistance gene detection. Clinicians were reminded that FA-PNEU^®^ does not detect all possible respiratory pathogens and should be interpreted alongside CC results and the overall clinical picture.

Empiric antibiotic therapy followed IDSA HAP/VAP recommendations, tailored to local epidemiology (including dual antipseudomonal coverage and anti-MRSA therapy when indicated). The decision to initiate, continue, escalate, or de-escalate antibiotics after FA-PNEU^®^ results was left to the discretion of the attending physician.

Demographic data, comorbidities, severity scores, prior antibiotic exposure, microbiological results, and antibiotic prescribing decisions were recorded in a dedicated database.

Because some patients contributed more than one respiratory sample during their ICU stay, each sample was analysed as an independent diagnostic episode. When multiple samples originated from the same patient, they were included independently for diagnostic performance analyses but clustered at the patient level for prescribing and outcome analyses. To account for potential within-patient correlation, antibiotic management outcomes were summarised per patient, while diagnostic metrics were calculated at the sample level with sensitivity analyses restricted to the first episode per patient to confirm consistency.

### 4.4. Outcomes

#### 4.4.1. Primary Outcome

- Change in antimicrobial management following availability of FA-PNEU^®^ results.
  ○ In patients not on antibiotics at testing: initiation vs. no initiation.
  ○ In patients already on antibiotics, we observed the following:
    ▪ No modification: same regimen continued.
    ▪ Escalation: addition of a new antibiotic or broadening of spectrum.
    ▪ De-escalation: discontinuation of an agent or narrowing of spectrum.

#### 4.4.2. Secondary Outcomes

1. Diagnostic performance of FA-PNEU^®^ compared with CC for organisms included in the panel, classifying results as follows:
  ○ True positive (TP): both FA-PNEU^®^ and CC positive for the same organism at the same semi-quantitative level.
  ○ True negative (TN): both negative.
  ○ False negative (FN): organism detected by CC but not by FA-PNEU^®^.
  ○ False positive (FP): organism detected by FA-PNEU^®^ but not by CC, excluding targets not expected to grow in culture.
2. Description of pathogens and resistance genes detected.

### 4.5. Statistical Analysis

Categorical variables will be expressed as counts and percentages; continuous variables as mean (standard deviation) or median (interquartile range) depending on distribution. Changes in antibiotic prescribing will be summarised descriptively. Agreement between FA-PNEU^®^ and CC will be assessed using Cohen’s kappa coefficient. Diagnostic accuracy metrics (sensitivity, specificity, PPV, NPV) will be calculated with 95% confidence intervals. Statistical analyses will be performed using R version 4.4.2, with *p* < 0.05 considered statistically significant.

## 5. Conclusions

FA-PNEU^®^ multiplex PCR had high diagnostic sensitivity and influenced antimicrobial management in more than half of suspected nosocomial LRTI episodes, often enabling earlier targeted therapy or the pursuit of alternative diagnoses. Its greatest clinical value lies in its ability to rapidly rule out bacterial pathogens and guide early stewardship interventions, but interpretation must remain anchored in comprehensive clinical and microbiological assessment.

## Figures and Tables

**Table 1:** Characteristics of the study population (according to patient-dependent variable).

Variable	Mean (SD)	n (%)
**Age in years**	62.68 (±0.12)	
**APACHE II**	20.86 (±0.33)	
**Male, n (%)**		158 (66.9)
**Reason for admission in ICU**		
Respiratory failure		80 (33.9)
Postoperative cardiac or vascular surgery		41 (17.4)
Septic shock		28 (11.9)
Intracranial bleeding		18 (7.6)
Postoperative general or urologic surgery		13 (5.5)
Depresses level of consciousness		13 (5.5)
Polytrauma		7 (3)
Traumatic brain injury		7 (3)
Cardiogenic shock		5 (2.1)
Postoperative neurosurgery		5 (2.1)
Others		19 (8)
**ICU mortality**		68 (28.8)

SD: standard deviation; APACHE II: Acute Physiology And Chronic Health Evaluation II; ICU: intensive care unit.

**Table 2:** Characteristics of the study population (according to sample-dependent variable). 344 samples.

Variable	n (%)	Mean (SD)	Median (IQR)
**Suspected nosocomial LRTI**			
HAP	170 (49.42)		
VAP	67 (19.48)		
VAT	107 (31.10)		
**Sample type, n (%)**			
Sputum	11 (3.2)		
Tracheal aspirate	45 (13.08)		
Bronchial aspirate	235 (68.31)		
BAL	52 (15.12)		
mini-BAL	1 (0.29)		
**SOFA score**		6.94 (±3.11)	
**Risk factors for multidrug-resistant bacteria,**	296 (86.04)		
**Days from hospital admission to multiplex PCR**			14 (20.25)
HAP			11 (20)
VAP			14 (23.5)
VAT			18 (17.5)
**Days from ICU admission to multiplex PCR**			8 (16)
HAP			6 (11)
VAP			15 (19.5)
VAT			9 (17.5)
**Prior antibiotic therapy before sample collection**			
No prior antibiotic	125 (36.34)		
Days of prior antibiotic			4 (5)

LRTI: Lower respiratory tract infections; HAP: Hospital-acquired pneumonia; VAP: Ventilator-associated pneumonia; VAT: Ventilator-associated tracheobronchitis; BAL: Bronchoalveolar lavage; mini-BAL: Mini-bronchoalveolar lavage; SOFA score: Sequential Organ Failure Assessment; IQR: interquartile range.

**Table 3:** Contingency table: Biofire^®^ Filmarray^®^ pneumonia panel plus multiplex polymerase chain reactive diagnostic performance compared to conventional culture.

	CC+	Negative CC−	Total
FA-PNEU^®^ +	57	99	156
FA-PNEU^®^ −	4	184	188
Total	61	283	344
FA-PNEU^®^		344 Samples	
Sensitivity (%)		93.4	
Specificity (%)		65	
PPV (%)		36.5	
NPV (%)		97.9	

FA-PNEU^®^: BioFire^®^ FilmArray^®^ Pneumonia Panel Plus; CC: conventional culture. PPV: Positive Predictive Value; NPV: Negative Predictive Value.

**Table 4:** Concordance Between FA-PNEU^®^ and Conventional Culture Results.

	344 Samples
**Complete concordance, n (%)**	196 (56.98)
**Incomplete concordance, n (%)**	148 (43.02)
Additional information provided by culture, n	23
Additional information provided by multiplex PCR, n	104
Additional information provided by culture and multiplex PCR, n	21

**Table 5:** Microorganisms included in the FA-PNEU^®^ panel: frequency of detection by multiplex PCR and conventional culture.

Microorganism	FA-PNEU^®^, N (%)	CC, N (%)
*Staphylococcus aureus*	48 (15.69)	22 (21.57)
*Haemophilus influenzae*	31 (10.13)	4(3.92)
*Serratia marcescens*	29 (9.48)	21 (20.59)
*Escherichia coli*	21 (6.86)	15 (14.71)
*Rhinovirus/Enterovirus*	21 (6.86)	0 (0)
*Streptococcus pneumoniae*	20 (6.54)	1 (0.98)
*Pseudomonas aeruginosa*	19 (6.21)	12 (11.76)
*Klebsiella pneumoniae group*	16 (5.23)	12 (11.76)
*Coronavirus*	15 (4.90)	0 (0)
*Enterobacter cloacae*	12 (3.92)	6 (5.88)
*Klebsiella oxytoca*	9 (2.94)	0 (0)
*Influenza A*	8 (2.61)	0 (0)
*Klebsiella aerogenes*	7 (2.29)	2 (1.96)
*Moraxella catarrhalis*	7 (2.29)	1 (0.98)
*Proteus *spp.	7 (2.29)	5 (4.90)
*Streptococcus agalactiae*	7 (2.29)	0 (0)
*Legionella pneumophila*	5 (1.63)	0 (0)
*Streptococcus pyogenes*	4 (1.31)	0 (0)
*Human parainfluenza virus*	4 (1.31)	0 (0)
*Respiratory syncytial virus*	4 (1.31)	0 (0)
*Acinetobacter calcoacetius–baumannii complex*	3 (0.98)	1 (0.98)
*Adenovirus*	3 (0.98)	0 (0)
*Mycoplasma pneumoniae*	2 (0.65)	0 (0)
*Influenza B*	2 (0.65)	0 (0)
*Metapneumovirus*	2 (0.65)	0 (0)
*Chlamydia pneumoniae*	0 (0)	0 (0)
*MERS-CoV*	0 (0)	0 (0)
Total	306 (100)	102 (100)

FA-PNEU^®^: BioFire^®^ FilmArray^®^ Pneumonia Panel Plus; CC: conventional culture.

**Table 6:** Antibiotic resistance mechanisms included in the FA-PNEU^®^ panel: frequency of detection by multiplex PCR and conventional culture.

Resistance Mechanism	FA-PNEU^®^, N (%)	CC, N (%)
*bla*_CTX-M_	3 (12.5)	2 (11.76)
*bla*_KPC_	4 (16.67)	3 (17.65)
*bla*_NDM_	0 (0)	0 (0)
*bla*_OXA-48 LIKE_	0 (0)	0 (0)
*bla*_VIM_	11 (45.83)	7 (41.18)
*bla*_IMP_	0 (0)	0 (0)
*bla*_mecA/mecC_ and *bla*_MREJ_	6 (25)	5 (29.41)
Total	24 (100)	17 (100)

**Table 7:** Microorganisms not included in the multiplex PCR Panel that grow on conventional media.

Microorganism	Detections (%)
*Aspergillus* spp.	12 (27.27)
*Stenotrophomonas maltophilia*	9 (20.45)
*Enterococcus* spp.	7 (15.91)
*Staphylococcus haemolyticus*	2 (4.55)
*Morganella morganii*	2 (4.55)
*Mycobacterium fortuitum*	1 (2.27)
*Staphylococcus epidermidis*	1 (2.27)
*Acinetobacter proteolyticus*	1 (2.27)
*Dolosigranulum pigrum*	1 (2.27)
*Klebsiella variicola*	1 (2.27)
*Sphingomonas paucimobilis*	1 (2.27)
*Streptococcus oralis*	1 (2.27)
*Staphylococcus hominis*	1 (2.27)
*Lactobacillus rhamnosus*	1 (2.27)
*Schaalia odontolytica*	1 (2.27)
Total	44 (100)

**Table 8:** Temporal distribution of analysed samples.

Year	Sampling Period (Months)	Number of Samples (n)	Monthly Average (Samples/Month)
2021	9	35	3.9
2022	12	81	6.8
2023	12	102	8.5
2024	12	118	9.8
2025	3	8	2.7

**Table 9:** Global and temporal distribution of antibiotic modification reasons.

Reason for Antibiotic Modification	GLOBAL	2021	2022	2023	2024	2025
	N (%)	N (%)	N (%)	N (%)	N (%)	N (%)
According to FA-PNEU^®^ results	198 (57.56)	19 (54.29)	47 (58.02)	58 (56.86)	69 (58.47)	5 (62.50)
Poor clinical evolution/severity	48 (13.95)	13 (37.14)	9 (11.11)	10 (9.80)	16 (13.56)	0 (0.00)
Suspected alternative infectious focus	61 (17.73)	3 (8.57)	20 (24.69)	16 (15.69)	19 (16.10)	3 (37.50)
Pending culture results	15 (4.36)	0 (0.00)	2 (2.47)	10 (9.80)	3 (2.54)	0 (0.00)
To cover colonizing organisms	4 (1.16)	0 (0.00)	2 (2.47)	1 (0.98)	1 (0.85)	0 (0.00)
Non-panel microorganisms	18 (5.23)	0 (0.00)	1 (1.23)	7 (6.86)	10 (8.47)	0 (0.00)
Total	344 (100)	35 (100)	81 (100)	102 (100)	118 (100)	8 (100)

FA-PNEU^®^: BioFire^®^ FilmArray^®^ Pneumonia Panel Plus.

**Table 10:** Real-world Modification of Antibiotic Therapy Following FA-PNEU^®^.

	Antibiotic Therapy Modification N (%)	According to FA-PNEU^®^ Results N (%)
**No prior antibiotic therapy before** **FA-PNEU^®^**	89 (25.87)	89 (100)
Initiation of antibiotic therapy after the multiplex PCR result	38 (11.05)	38 (100)
No initiation of antibiotic therapy after the multiplex PCR result	51 (14.82)	51 (100)

FA-PNEU^®^: BioFire^®^ FilmArray^®^ Pneumonia Panel Plus.

## Data Availability

The raw data supporting the conclusions of this article will be made available by the authors on request.

## Abbreviations

The following abbreviations are used in this manuscript:

LRTI	Lower respiratory tract infections
FA-PNEU^®^	BioFire^®^ FilmArray^®^ Pneumonia Panel Plus
HAP	Hospital-acquired pneumonia
VAP	Ventilator-associated pneumonia
VAT	Ventilator-associated tracheobronchitis
CC	Conventional culture
MRSA	Methicillin-resistant Staphylococcus aureus
IDSA	Infectious Diseases Society of America
RS	Respiratory secretion
CAP	Community-acquired pneumonia
SD	Standard deviation
APACHE II	Acute Physiology And Chronic Health Evaluation II
ICU	Intensive care unit
BAL	Bronchoalveolar lavage
Mini-BAL	Mini-bronchoalveolar lavage
SOFA score	Sequential Organ Failure Assessment score
IQR	Interquartile range
PPV	Positive Predictive Value
NPV	Negative Predictive Value
TP	True positive
TN	True negative
FN	False negative
FP	False positive
